# The Association of Sleep Trouble and Physical Inactivity with Breast Cancer Risk in Nova Scotia: Evidence from the Atlantic PATH Cohort

**DOI:** 10.3390/ijerph22040471

**Published:** 2025-03-22

**Authors:** Cindy Feng, Ellen Sweeney

**Affiliations:** Department of Community Health and Epidemiology, Faculty of Medicine, Dalhousie University, Halifax, NS B3H 1V7, Canada; ellen.sweeney@dal.ca

**Keywords:** breast cancer incidence, sleep trouble, physical activity, modifiable risk factors, Atlantic PATH cohort

## Abstract

Breast cancer is a major public health concern, and modifiable health behaviors such as sleep quality and physical activity may influence risk. This study examined the associations between self-reported sleep trouble, sleep duration, and physical activity with breast cancer incidence in a prospective longitudinal cohort of 10,305 females from Nova Scotia. Breast cancer cases were identified through record linkage to the Nova Scotia Cancer Registry. Multivariable logistic regression models were used to estimate adjusted odds ratios (AORs) and 95% confidence intervals (CIs), accounting for sociodemographic factors, reproductive history, comorbidities, and other health behaviors. Frequent sleep trouble (“all of the time”) was significantly associated with increased odds of breast cancer (AOR = 2.41, 95% CI = 1.09–5.34, *p* = 0.03), while no significant associations were observed between sleep duration and breast cancer risk. High physical activity was significantly associated with a lower risk of breast cancer (AOR = 0.58, 95% CI = 0.39–0.86, *p* < 0.01). These findings suggest that frequent sleep disturbances may be associated with an increased risk of breast cancer, while high physical activity appears to be linked to a lower risk of breast cancer. Further research is needed to explore these relationships and their underlying mechanisms.

## 1. Introduction

Breast cancer is the most frequently diagnosed cancer and a leading cause of cancer-related mortality among women worldwide, including in Canada [1]. While genetic predispositions and hormonal influences contribute significantly to breast cancer risk, modifiable lifestyle factors are increasingly recognized as important considerations for cancer prevention [2]. Among these factors, sleep behavior [3,4] and physical activity [5] have gained considerable attention for their potential roles in carcinogenesis, though the evidence regarding their precise impact, particularly for sleep quality, remains complex and sometimes controversial [6].

Sleep quality is commonly defined as an individual’s self-satisfaction with all aspects of the sleep experience, including attributes such as sleep efficiency, sleep latency, sleep duration, and wake after sleep onset [7]. In this study, we focus on two components often used in epidemiological research: sleep troubles and sleep duration. While these measures may not capture the full range of sleep quality, they were selected due to data availability in the current study and are widely utilized in studies where more comprehensive assessments, such as polysomnography or actigraphy, are not available.

Sleep trouble or disturbance, characterized by difficulty initiating or maintaining sleep, has been implicated in various health conditions, including cardiovascular diseases [8,9], metabolic disorders [10], and impaired cognitive performance [11]. Emerging evidence highlights links between sleep problems, disrupted circadian rhythms, and cancer risk [3,4]. Although the underlying mechanisms remain unclear, proposed biological mechanisms include circadian dysregulation, altered melatonin secretion, heightened inflammation, and oxidative stress, all of which may promote carcinogenesis [12]. Despite these associations, the literature is mixed; some studies report significant links between sleep disturbance and breast cancer risk, while others find no such relationship [13,14,15].

Sleep duration, typically measured as the total hours slept per night, has also been extensively studied concerning cancer risk [16]. However, findings specific to breast cancer remain inconsistent. Some studies suggest that insufficient sleep (<6 h per night) and excessive sleep (>9 h per night) are associated with increased breast cancer risk, potentially due to hormonal dysregulation involving estrogen and progesterone [17]. Conversely, other studies report no significant associations [13,14,18,19], while some have shown either adverse [17] or protective effects of long sleep duration on breast cancer risk [20]. These inconsistencies underscore the complexity of the relationship between sleep duration and breast cancer risk, further highlighting the need for ongoing investigation.

Physical activity is a well-documented modifiable factor in chronic disease prevention and has been extensively studied in relation to cancer risk [21]. Numerous studies suggest that increased physical activity may reduce the risk of breast cancer, potentially through mechanisms such as hormonal regulation, reduced inflammation, and enhanced immune function [5,21,22,23,24,25]. While the evidence consistently indicates a protective effect, some variability exists, with certain studies highlighting significant benefits, particularly for vigorous or high levels of activity, while others report no associations or suggest a threshold effect [26]. These variations underscore the need for further research to elucidate the role of physical activity in breast cancer prevention, particularly across diverse populations and activity patterns.

Despite considerable research on these factors individually, few studies have simultaneously examined the independent associations of sleep disturbance, sleep duration, and physical activity with breast cancer risk within a single cohort. Furthermore, there is a lack of research focusing on Canadian populations, particularly those in the Atlantic provinces. This study aimed to address these gaps by investigating the relationships between sleep troubles, sleep duration, physical activity, and subsequent breast cancer incidence in a prospective longitudinal cohort of cancer-free females in Nova Scotia, using linked provincial cancer registry data.

## 2. Materials and Methods

### 2.1. Study Design

This study is based on data from the Atlantic Partnership for Tomorrow’s Health (Atlantic PATH) study [27], which is part of the larger Canadian Partnership for Tomorrow’s Health (CanPath) [28]. CanPath is a multi-center, prospective cohort study with more than 330,000 participants aimed at investigating the influence of behavioral, environmental, and genetic factors on the development of cancer and other chronic diseases [28]. The Atlantic PATH cohort specifically includes residents from the four provinces of Atlantic Canada: Nova Scotia, New Brunswick, Prince Edward Island, and Newfoundland and Labrador.

### 2.2. Study Setting

The Atlantic PATH cohort consists of more than 34,000 participants aged 30–74 years who provided informed consent between 2009 and 2015 as part of their enrollment in the Atlantic PATH study. Participants were recruited through a range of outreach activities, including invitations from the Provincial Health Insurance provider (Nova Scotia only), advertising, media coverage, community and workplace events, incentive programs (e.g., Airmiles), and community champions who encouraged participation. No specific probabilistic sampling method was employed. Participants completed a self-administered questionnaire to collect a comprehensive set of variables, including demographic information, lifestyle factors, health behaviors, and medical history [27]. Despite the diverse recruitment methods, all participants provided informed consent for the use of their data in health research [27]. For the current analysis, we focused on data from Nova Scotia, where provincial cancer registry data, which are population-based, have been linked to the cohort through December 2020, enabling an in-depth examination of cancer incidence.

### 2.3. Participants

To ensure the inclusion of only those who were cancer-free at baseline, we cross-referenced each participant’s baseline age with the age at diagnosis recorded in the cancer registry. Participants diagnosed with cancer prior to enrollment were excluded, ensuring the cohort consisted solely of cancer-free females at the time of enrollment. Additionally, participants who were diagnosed with any cancer other than breast cancer during follow-up were also excluded from the analysis.

### 2.4. Ethical Approval

This secondary analysis of Atlantic PATH data was approved by the Health Sciences Research Ethics Board at Dalhousie University (REB#: 2022-6114).

### 2.5. Variables

#### 2.5.1. Exposure Variables

Sleep Trouble: Participants were asked about the frequency of sleep trouble, including difficulties falling asleep or staying asleep. Response options ranged from 0 (none of the time) to 4 (all of the time). This variable was treated as a five-level categorical variable.

Sleep Duration: Sleep duration was self-reported as the total hours of sleep, including naps, typically obtained each day. Originally categorized into 12 groups (ranging from less than 3 h to 12 or more hours), the variable was collapsed into five broader categories due to small sample sizes: <6 h, 6 to <7 h, 7 to <8 h, 8 to <9 h, and ≥9 h.

Physical Activity: Physical activity was assessed using the short or long versions of the International Physical Activity Questionnaire (IPAQ) [29] and categorized into three levels (low, moderate, and high) according to IPAQ scoring guidelines.

#### 2.5.2. Outcome Variable

Breast cancer incidence was the primary outcome. Participants were followed through record linkage to the Nova Scotia Cancer Registry, which provided information on cancer diagnoses through December 2020. Cancer diagnoses were coded using the International Classification of Diseases for Oncology (ICD-O-3) topography codes. Breast cancer was defined as the first primary case of invasive or ductal carcinoma in situ (DCIS) (ICD-O-3: C50.0–C50.9). Only the first instance of breast cancer was considered in the analysis; subsequent diagnoses or recurrences were excluded.

#### 2.5.3. Covariates

Several covariates were included to account for potential confounding.

Sociodemographic and Economic Factors: *Age*: Age at baseline enrollment was treated as a continuous variable (years). *Ethnicity*: Ethnicity was categorized into broad groups (e.g., Caucasian, Indigenous, South Asian, and East Asian). A binary variable contrasting those of exclusively European descent with those reporting at least partial non-European ancestry was created, given that 88.87% of participants were of European descent. *Education*: Education level (highest degree attained) was categorized into four groups: high school or lower, community college/trades, university undergraduate/certificate, and university graduate education.

Medical and Reproductive History: *Menopausal Status*: Menopausal status was derived from baseline menstrual history (still menstruating/ceased menstruating). *Age at Menarche*: Age at menarche (years) was analyzed as a continuous variable. *Number of Live Births*: The total number of live births was treated as a continuous variable. *Family History of Breast Cancer*: Family history of breast cancer (yes/no) indicated whether a first-degree relative had been diagnosed with breast cancer. *Hormone Replacement Therapy (HRT) Use*: Ever use of HRT (yes/no) was assessed.

Health Behaviors: *Smoking Status*: Smoking status was categorized as follows: never smoked, past smoker (smoked ≥100 cigarettes but no longer smokes), current occasional smoker, and current daily smoker. *Alcohol Consumption*: Alcohol consumption was measured based on the frequency of weekly alcohol intake during the past 12 months (never, less than once a month, about once a month, 2 to 3 times a month, once a week, 2 to 3 times a week, 4 to 5 times a week, and 6 to 7 times a week).

### 2.6. Statistical Methods

Descriptive statistics were used to summarize the study population, stratified by breast cancer onset status. The number of complete respondents (excluding missing values) for each variable is indicated in brackets in the first column of Table 1. Categorical variables were presented as frequencies and percentages, while continuous variables were summarized as medians with interquartile ranges (IQRs) (Table 1).

To address missing data, multiple imputation by chained equations (MICE) [30] was employed prior to conducting any inferential analyses. MICE was chosen due to its flexibility in handling mixed data types and its ability to account for uncertainty in missing data. Twenty imputed datasets were generated, incorporating all variables listed in Table 1 as predictors in the imputation model. Rubin’s rules were applied to combine parameter estimates and standard errors across the imputed datasets, ensuring robust and unbiased results [30].

Bivariate logistic regression analyses were performed to evaluate the unadjusted relationships between each covariate and breast cancer incidence. Odds ratios (ORs) with 95% confidence intervals (CIs) were calculated. Variables with a *p*-value ≤ 0.25 in the bivariate analyses were considered for inclusion in the multivariable model. This less stringent threshold was chosen to reduce the risk of excluding potential confounders in the multivariable analysis.

Multivariable logistic regression models were then constructed to estimate adjusted odds ratios (AORs) and 95% CIs for the associations between sleep trouble, sleep duration, physical activity, and breast cancer incidence, adjusting for the selected covariates. Backward stepwise selection was used to refine the multivariable model, with a removal criterion of *p* > 0.05. Interaction terms between each exposure variable (sleep trouble, sleep duration, and physical activity) and each covariate included in the final multivariable model were tested individually to identify potential effect modification. All statistical analyses were conducted using Stata/SE 18.

## 3. Results

The study cohort, as described in Table 1, included 10,305 participants, with 213 (2.1%) developing breast cancer. Breast cancer incidence was higher among postmenopausal females (2.64%) compared to premenopausal females (1.46%). Incidence was also higher among those with a family history of breast cancer (3.67% vs. 1.99% among those without a family history) and users of hormone replacement therapy (3.02% vs. 1.86% among non-users). The majority of the cohort were of European descent (88.87%), with a slightly higher incidence observed among those with a high school education or lower (2.63%) compared to those with higher levels of education (1.87–1.92%). Health behaviors showed varying trends; past smokers had a higher incidence of breast cancer (2.66%) compared to never smokers (1.5%), while current occasional smokers had the highest incidence rate (2.82%). Those with low physical activity had a higher incidence (2.71%) compared to those with moderate (1.97%) or high (1.83%) physical activity. Sleep patterns revealed that participants reporting sleep trouble “all of the time” had the highest incidence (3.09%) compared to those reporting “none of the time” (1.38%). Similarly, those sleeping less than 6 h (1.80%) or between 7 and 8 h (2.10%) had a slightly higher incidence compared to other sleep durations. While Table 1 provides an overview of the distribution of variables, statistical significance was assessed through bivariate analyses, as summarized in Table 2.

Table 2 presents the results of the bivariate analysis examining associations between potential covariates and breast cancer onset. Several variables met the pre-specified criterion for inclusion in the multivariable model (*p* < 0.25), including age, education, family history of breast cancer, menopausal status, age at menarche, history of HRT usage, comorbidity, smoking status, sleep trouble, and physical activity. Although the association between sleep duration and breast cancer onset did not reach this threshold (*p* > 0.25), sleep duration was retained in the multivariable model because it was a primary exposure of interest.

Table 3 summarizes the multivariable logistic regression results examining the relationship between sleep trouble, sleep duration, physical activity, and breast cancer onset, adjusted for control variables after backward selection. No statistically significant interactions were observed between any of the exposure variables (sleep trouble, sleep duration, and physical activity) or between any exposure variable and the other covariates included in the model. Participants reporting sleep trouble “all of the time” had significantly higher odds of developing breast cancer compared to those reporting “none of the time” (AOR = 2.41, 95% CI = 1.09–5.34, *p* = 0.03). Other levels of sleep trouble, such as “a little of the time”, “some of the time”, and “most of the time”, were not significantly associated with breast cancer onset. Sleep duration was not significantly associated with breast cancer onset across any category. Adjusted odds ratios ranged from 1.14 to 1.71 when compared to the reference group (<6 h), with *p*-values exceeding 0.1, indicating no strong evidence of an association. For physical activity, high physical activity was significantly associated with lower odds of breast cancer onset compared to low physical activity (AOR = 0.58, 95% CI = 0.39–0.86, *p* < 0.01).

Several covariates showed significant associations, including age, family history of breast cancer, and past smoking. Each additional year of age was associated with a 5% increase in breast cancer risk (AOR = 1.05, 95% CI = 1.03–1.07, *p* < 0.01). A family history of breast cancer significantly increased the odds of disease onset (AOR = 1.80, 95% CI = 1.07–3.00, *p* = 0.03). Compared to never smokers, past smokers had significantly higher odds of breast cancer onset (AOR = 1.67, 95% CI = 1.23–2.26, *p* < 0.01), while current occasional smokers showed a marginal association (AOR = 2.17, 95% CI = 0.98–4.81, *p* = 0.06). Current daily smoking was not significantly associated with breast cancer onset. Interestingly, participants with more than three comorbidities had lower odds of developing breast cancer compared to those with no comorbidities (AOR = 0.29, 95% CI = 0.10–0.82, *p* = 0.02).

## 4. Discussion

Our finding that *persistent* sleep trouble (“all the time”) significantly increases breast cancer risk is consistent with emerging evidence indicating that *severe* sleep disturbances may play a crucial role in cancer development. Sleep disturbance has been linked to several biological pathways that may contribute to cancer risk. One proposed mechanism is the impairment of immune function, where chronic sleep disturbances may weaken the body’s ability to detect and eliminate cancerous cells [11,14,31]. Another plausible pathway involves metabolic dysregulation [32,33], which can lead to obesity, a well-established risk factor for breast cancer. Chronic sleep disruption has been shown to affect hormones like leptin and ghrelin, which regulate appetite and energy balance, potentially promoting weight gain and metabolic changes conducive to cancer development [34]. An additional mechanism involves alterations in melatonin release. Melatonin, a hormone regulated by the light/dark cycle, has antioxidant and anti-carcinogenic properties [35]. Disruptions in the circadian rhythm, often associated with sleep disturbances, may reduce melatonin secretion, thereby increasing cancer risk [36]. While these pathways—impaired immune function, metabolic dysregulation, and altered melatonin release—have varying levels of supporting evidence, the precise biological mechanisms linking sleep disturbances to cancer risk remain incompletely understood [37]. Further research is needed to delineate these mechanisms and establish the extent to which persistent sleep trouble contributes to breast cancer risk.

Regarding physical activity, our findings reaffirm its protective effects against breast cancer, consistent with extensive evidence in the literature. Numerous studies have demonstrated an inverse relationship between physical activity and breast cancer risk, with proposed mechanisms including reductions in systemic inflammation, improved immune function, hormonal regulation, and enhanced insulin sensitivity [2,5,21,23,26,38]. Notably, in our study, the relationship between physical activity and breast cancer risk was strongest at higher activity levels, aligning with research suggesting that higher levels of physical activity provide greater protection [23,38,39]. This suggests that exceeding standard physical activity recommendations might yield added benefits for breast cancer prevention. Further research is needed to better understand this relationship, particularly the role of activity intensity, frequency, and duration in modifying risk. Studies incorporating objective measures of physical activity, such as accelerometers, could provide more accurate insights into the specific thresholds needed for risk reduction. Additionally, investigating biological mediators—such as adiposity, inflammatory markers, and sex hormones—could help elucidate the pathways through which physical activity exerts its protective effects. These findings could inform tailored recommendations for physical activity that optimize breast cancer prevention strategies across different populations.

We also investigated potential interactions between sleep disturbance and physical activity, sleep disturbance and sleep duration, and physical activity and sleep duration in relation to breast cancer risk. However, no statistically significant interactions were observed. This lack of significant interaction could be due to a true absence of interaction, limited statistical power (particularly given the relatively low number of breast cancer events), or measurement error in our self-reported exposure data.

## 5. Strengths and Limitations

This study has several notable strengths. First, it utilized a large, longitudinal cohort from the Atlantic PATH study, providing a foundation for examining long-term health outcomes. Importantly, the study employed comprehensive follow-up through linkage with cancer registry data, ensuring reliable and accurate breast cancer incidence ascertainment, which is essential for precise outcome measurement. The inclusion of detailed covariate adjustment for various known risk factors helped minimize potential confounding, enhancing the validity of the observed associations. Furthermore, the focus on modifiable health behaviors, such as sleep trouble, sleep duration, and physical activity, provides actionable insights for breast cancer prevention. These behaviors are particularly relevant because they are amenable to intervention, offering potential public health benefits if further research confirms their relationship with breast cancer risk. Additionally, the use of multiple imputation to address missing data strengthened the analysis by reducing potential bias and increasing statistical power.

Despite these strengths, several limitations should be considered. One key limitation stems from the absence of death or out-migration data. Some individuals may have moved out of the province or died from other causes before a cancer diagnosis, meaning they would not be recorded in the cancer registry despite potentially developing breast cancer elsewhere. Without these data, we cannot account for these potential events, which could lead to an underestimation of breast cancer incidence, as these individuals would contribute to the denominator (total at-risk population) but not the numerator (number of cases). Another limitation is the reliance on baseline data for health behaviors such as sleep trouble, sleep duration, and physical activity, without considering changes in these behaviors over time. Health behaviors may evolve, and without capturing these changes, we cannot assess their long-term impact on breast cancer risk. This may lead to underestimation or misinterpretation of the effects of these behaviors. Another limitation is the relatively short follow-up period (baseline data collected between 2009 and 2015, with cancer registry data linked until December 2020). Because breast cancer can take many years to develop, this limited follow-up time may have prevented us from observing the full long-term impact of the exposures on breast cancer risk, particularly for slower-developing cancers.

Potential selection bias is also a consideration. Participants in the Atlantic PATH cohort may differ from non-participants in ways that could influence the generalizability of our findings. For example, individuals who are healthier or more health-conscious might be more likely to participate, potentially leading to an underestimation of the true associations between exposures and breast cancer risk. The predominantly European-ancestry cohort may also limit the generalizability of the findings to more diverse populations. Additionally, self-reported data on sleep trouble, sleep duration, and physical activity are subject to recall bias and misclassification. These measurement errors, which are likely non-differential, could attenuate the observed associations, making them appear weaker than they actually are. While the study adjusted for several known confounders, residual confounding may still be present due to unmeasured factors such as genetic predispositions.

We acknowledge that the relatively low incidence of breast cancer in our cohort (213 cases out of 10,305 participants) limits the ability to detect very small effects. Our final multivariable model includes 19 parameters (18 regression coefficients and 1 intercept). The events per variable (EPV) rule, which suggests a minimum of 10–20 events per parameter for stable and reliable estimates [40,41], implies that at least 190–380 events are desirable. With 213 breast cancer cases, our study satisfies the lower bound of this guideline. However, we recognize that EPV alone does not guarantee the adequacy of a model, as emerging research suggests that factors such as model complexity, collinearity, and predictor distributions also influence estimation reliability [42]. To assess our study’s ability to detect meaningful associations, we conducted a post hoc power analysis using G*Power [43]. Assuming a logistic regression model, we tested the ability to detect an odds ratio of 2.41 for the association between sleep trouble (“all the time” vs. “none of the time”) and breast cancer incidence. The analysis was based on the observed breast cancer incidence rate of 1.38% in the “none of the time” category, the prevalence of the “all the time” category (5.61%), a total sample size of 10,305 participants, and an R^2^ of 0.1 for other covariates. The power analysis indicated that the study had a statistical power of 84.47%, exceeding the commonly accepted 80% threshold for detecting meaningful effects. However, we acknowledge the ongoing debate surrounding post hoc power analysis, particularly its reliance on observed data, potential for circular reasoning, and limited utility in assessing study validity [44,45]. While our large sample size provides some advantages, the low event rate may still limit the statistical power to detect smaller but potentially important associations—especially in subgroup analyses or when examining less common exposures or interactions. This limitation is particularly relevant to the non-significant findings for certain levels of sleep trouble and sleep duration, where a true association may exist but remain undetected due to insufficient power. Therefore, these null findings should be interpreted with caution.

Moreover, lifestyle factors such as physical activity and sleep troubles can influence the characteristics of breast cancer tumors in various ways. These factors may affect tumor behavior through mechanisms like inflammation, hormone levels, immune function, and the tumor microenvironment. For example, physical activity has been linked to reduced inflammation and improved immune function [5,21,22,23,24,25], while sleep troubles may affect hormone levels and contribute to chronic inflammation, potentially influencing tumor progression [12]. While our study highlights the importance of considering these factors, we were unable to directly assess the biological mechanisms underlying their effects. Future research should further explore how physical activity and sleep troubles influence the progression of specific tumor and clinical characteristics to inform personalized treatment and prevention strategies.

## 6. Conclusions

In conclusion, this study found that persistent sleep trouble (“all the time”) was statistically significantly associated with an increased risk of breast cancer in the Nova Scotia component of the Atlantic PATH cohort, while high levels of physical activity were statistically significantly associated with a reduced risk. These findings, from a large prospective cohort with comprehensive follow-up through cancer registry linkage, contribute to the growing body of evidence highlighting the potential role of modifiable lifestyle factors in breast cancer risk and prevention. While several limitations, including the relatively short follow-up, reliance on baseline self-reported data, and potential residual confounders, should be considered, our study offers valuable insights into how sleep and physical activity may be linked to breast cancer risk. Future research should focus on longer follow-up studies with objective measures of sleep and physical activity, incorporating genetic and other relevant data, to further explore the complex relationships between lifestyle factors and breast cancer risk.

## Figures and Tables

**Table 1:** Characteristics of the study cohort, including sociodemographic, health behavior, and medical history factors. **Note:** Categorical variables are presented as n (%), with the number in brackets representing column percentages for the total cohort and row percentages for breast cancer onset (Yes/No). Continuous variables, including *age*, *age at menarche*, *and number of births*, are presented as medians (IQRs).

Characteristics	Category		Breast Cancer Onset	
		Total n (%)	Yes n (%)	No n (%)
		10,305 (100)	213 (2.1)	10,092 (97.93)
**Sociodemographic and Economic Status**				
Age (n = 10,305)		52 (15)	57 (13)	52 (15)
Ethnicity (n = 10,305)	European descendant only	9158 (88.87)	192 (2.10)	8966 (97.90)
	Non-European descendant	1147 (11.13)	21 (1.83)	1126 (98.17)
Education (completed highest) (n = 9791)	High school or lower	1671 (17.07)	44 (2.63)	1627 (97.37)
	Trades or community college	3458 (35.32)	65 (1.88)	3393 (98.12)
	University certificate	3155 (32.22)	59 (1.87)	3096 (98.13)
	Graduate degree	1507 (15.39)	29 (1.92)	1478 (98.08)
**Medical and Reproductive History**				
Family history of breast cancer (n = 10,305)	Yes	463 (4.49)	17 (3.67)	446 (96.33)
	No	9842 (95.51)	196 (1.99)	9646 (98.01)
Menopausal status (n = 10,305)	Postmenopausal	5312 (51.55)	140 (2.64)	5172 (97.36)
	Premenopausal	4993 (48.45)	73 (1.46)	4920 (98.54)
Age at menarche (n = 9497)		13 (2)	13 (2)	13 (2)
Number of births (n = 9478)		2 (1)	2 (1)	2 (1)
History of HRT usage (n = 8727)	Yes	2019 (23.14)	61 (3.02)	1958 (96.98)
	No	6708 (76.86)	125 (1.86)	6583 (98.14)
Comorbidity (total number of diseases) (n = 10,305)	0	3870 (37.55)	76 (1.96)	3794 (98.04)
	1	3385 (32.85)	68 (2.01)	3317 (97.99)
	2–3	2674 (25.95)	65 (2.43)	2609 (97.57)
	Above 3	376 (3.65)	4 (1.06)	372 (98.94)
**Health Behaviors**				
Smoking status (n = 9749)	Never smoked	5131(52.63)	77 (1.5)	5054 (98.50)
	Past smoker	3647 (37.41)	97 (2.66)	3550 (97.34)
	Current occasional smoker	248 (2.54)	7 (2.82)	241 (97.18)
	Current daily smoker	723 (7.42)	14 (1.94)	709 (98.06)
Alcohol consumption (n = 9770)	Never	904 (9.25)	17 (1.88)	877 (98.12)
	Less than once a month	2393 (24.49)	49 (2.05)	2344 (97.95)
	About once a month	852 (8.72)	15 (1.76)	837 (98.24)
	2 to 3 times a month	1405 (14.38)	39 (2.78)	1366 (97.22)
	Once a week	1182 (12.10)	19 (1.61)	1163 (98.39)
	2 to 3 times a week	1654 (16.93)	33 (2.00)	1621 (98.00)
	4 to 5 times a week	774 (7.92)	11 (1.42)	763 (98.58)
	6 to 7 times a week	606 (6.20)	15 (2.48)	591 (97.52)
Sleep trouble (n = 9808)	None of the time	798 (8.14)	11 (1.38)	787 (98.62)
	A little of the time	2726 (27.79)	53 (1.94)	2673 (98.06)
	Some of the time	3943 (40.20)	78 (1.98)	3865 (98.02)
	Most of the time	1791 (18.26)	37 (2.07)	1754 (97.93)
	All the time	550 (5.61)	17 (3.09)	533 (96.91)
Sleep duration (n = 9659)	<6	667 (6.91)	12 (1.80)	655 (98.20)
	[6, 7)	1849 (19.14)	37 (2.00)	1812 (98.00)
	[7, 8)	3570 (36.96)	75 (2.10)	3495 (97.90)
	[8, 9)	2891 (29.93)	59 (2.04)	2832 (97.96)
	≥9	682 (7.06)	10 (1.47)	672 (98.53)
Physical activity (n = 7280)	Low	1401 (19.24)	38 (2.71)	1363 (97.29)
	Moderate	2432 (33.41)	48 (1.97)	2384 (98.03)
	High	3447 (47.35)	63 (1.83)	3384 (98.17)

**Table 2:** Bivariate analysis of associations between sociodemographic, health behavior, and medical factors with breast cancer onset in the study cohort (n = 10,305).

			Breast Cancer Onset		
		OR	95% CI		*p*-Value
			Lower	Upper	
**Sociodemographic and Economic Status**					
Age		1.05	1.03	1.06	<0.01
Ethnicity (Ref: Non-European descendant)	European descendant only	1.15	0.73	1.81	0.55
Education (Ref: High school or lower)	Trades or community college	0.70	0.47	1.04	0.07
	University certificate	0.69	0.47	1.02	0.06
	Graduate degree	0.70	0.44	1.12	0.14
**Medical and Reproductive History**					
Family history of breast cancer (Ref: No)	Yes	1.88	1.13	3.11	0.02
Menopausal status (Ref: Premenopausal)	Postmenopausal	1.82	1.37	2.43	<0.01
Age at menarche		1.07	0.97	1.18	0.17
Number of births		0.96	0.85	1.08	0.47
History of HRT usage (Ref: No)		1.70	1.26	2.30	<0.01
Comorbidity (Ref: None)	1	1.02	0.74	1.42	0.89
	2–3	1.24	0.89	1.74	0.20
	Above 3	0.54	0.20	1.48	0.23
**Health Behaviors**					
Smoking status (Ref: Never smoked)	Past smoker	1.82	1.35	2.46	<0.01
	Current occasional smoker	1.97	0.90	4.34	0.09
	Current daily smoker	1.34	0.77	2.33	0.30
Alcohol consumption (Ref: Never)	Less than once a month	1.09	0.62	1.92	0.77
	About once a month	0.91	0.45	1.84	0.79
	2 to 3 times a month	1.48	0.83	2.66	0.19
	Once a week	0.83	0.43	1.58	0.57
	2 to 3 times a week	1.03	0.57	1.88	0.91
	4 to 5 times a week	0.74	0.35	1.60	0.45
	6 to 7 times a week	1.31	0.65	2.64	0.46
Sleep trouble (Ref: None of the time)	A little of the time	1.36	0.71	2.59	0.35
	Some of the time	1.36	0.72	2.56	0.34
	Most of the time	1.41	0.71	2.78	0.33
	All of the time	2.34	1.10	4.98	0.03
Sleep duration (Ref: <6)	[6, 7)	1.10	0.57	2.13	0.78
	[7, 8)	1.14	0.62	2.09	0.66
	[8, 9)	1.12	0.60	2.09	0.72
	≥9	0.82	0.35	1.93	0.65
Physical activity (Ref: Low)	Moderate	0.68	0.45	1.05	0.08
	High	0.62	0.42	0.92	0.02

**Table 3:** Multivariable logistic regression analysis of the association between sleep trouble, sleep duration, physical activity, and breast cancer onset, adjusted for sociodemographic, reproductive, and health behavior covariates (n = 10,305).

	AOR	95% CI		*p*-Value	Sig
Sleep trouble (Ref: None of the time)					
A little of the time	1.24	0.65	2.38	0.51	
Some of the time	1.18	0.62	2.24	0.61	
Most of the time	1.29	0.64	2.60	0.47	
All the time	2.41	1.09	5.34	0.03	**
Sleep duration (Ref: <6)					
[6, 7)	1.49	0.75	2.94	0.25	
[7, 8)	1.71	0.89	3.26	0.11	
[8, 9)	1.62	0.83	3.17	0.16	
≥9	1.14	0.47	2.74	0.78	
Physical activity (Ref: Low)					
Moderate High	0.67	0.43	1.03	0.07	*
	0.58	0.39	0.86	<0.01	***
Age	1.05	1.03	1.07	<0.01	***
Family history of breast cancer (Ref: No)	1.77	1.07	3.00	0.03	**
Smoking status (Ref: Never smoked)					
Past smoker	1.67	1.23	2.26	<0.01	***
Current occasional smoker	2.17	0.98	4.81	0.06	*
Current daily smoker	1.40	0.80	2.45	0.24	
Comorbidity (Ref: None)					
1	0.85	0.61	1.19	0.33	
2–3	0.84	0.59	1.19	0.32	
Above 3	0.29	0.10	0.82	0.02	**

*** *p* < 0.01, ** *p* < 0.05, * *p* < 0.1.

## Data Availability

Data and biosamples from Atlantic PATH are available to researchers through a data access process. Additional information can be obtained by contacting info@atlanticpath.ca.
